# Effects of heavy metal exposure during pregnancy on birth outcomes

**DOI:** 10.1038/s41598-023-46271-0

**Published:** 2023-11-03

**Authors:** Sabrina Shafi Zinia, Ki-Hyeok Yang, Eun Ju Lee, Myoung-Nam Lim, Jeeyoung Kim, Woo Jin Kim, Ko-CHENS Study group, Choonghee Park, Hyun Jeong Kim, Soon-Won Jung, Sooyeon Hong, A-Ra Jung, Jueun Lee, Seung Do Yu, Namkyoung Hwang, Dong Jin Jeong, Heung Won Seo, Hae Soon Kim, Myeongjee Lee, Eun Kyo Park, Seulbi Lee, Hoon Kook, Hee Jo Baek, Jai Dong Moon, Won Ju Park, Myung-Geun Shin, Ki-Chung Paik, Ho-Jang Kwon, Myung-Ho Lim, Seung Jin Yoo, Sanghyuk Bae, Young-Seoub Hong, Yu-Mi Kim, Jeong-Wook Seo, Myo Jing Kim, Hee Won Chueh, Dae Hyun Lim, Jeong Hee Kim, Joohye Park, Donghyun Kim, Hye Ju So, Sung-Chul Hong, Keun Hwa Lee, Su-Young Kim, Sunghun Na, Ji Tae Choung, Young Yoo, Sung Chul Seo, Hyeonju Kang, Ji Yeon Jang, Minyoung Jung, Se-Jin Chun, Young-Min Kim, Jihyun Kim, Youn-Hee Lim, Joong Shin Park, Chan-Wook Park, Choong Ho Shin, Kuck Hyeun Woo, SungYong Choi, Jin Kyung Kim, Wonho Yang, Jongil Hur, Myung-Sook Park, Kyung-Hwa Choi, Seung-Hwa Lee, Inbo Oh, Jiho Lee, Chang Sun Sim

**Affiliations:** 1 Department of Internal Medicine and Environmental Health Center, School of Medicine, Kangwon National University, Chuncheon, 24341 Republic of Korea; 2 Department of Environmental Health Research, Environmental Health Research Division, National Institute of Environmental Research, Ministry of Environment, Incheon, Republic of Korea; 3 Environmental Health Policy Division, Office of Environmental Health, Ministry of Environment, Sejong, Republic of Korea; 4 Department of Pediatrics, Ewha Womans University Medical Center, Seoul, Republic of Korea; 5 Department of Occupational and Environmental Medicine, School of Medicine, Ewha Womans University, Seoul, Republic of Korea; 6 Department of Medical Science, School of Medicine, Ewha Womans University, Seoul, Republic of Korea; 7 Environmental Health Center for Childhood Leukemia and Cancer, and Department of Pediatrics, Chonnam National University Hwasun Hospital, Hwasun, Republic of Korea; 8 Environmental Health Center for Childhood Leukemia and Cancer, and Department of Occupational and Environmental Medicine, Chonnam National University Hwasun Hospital, Hwasun, Republic of Korea; 9 Environmental Health Center for Childhood Leukemia and Cancer, and Department of Laboratory Medicine, Chonnam National University Hwasun Hospital, Hwasun, Republic of Korea; 10 Department of Psychiatry, Dankook University College of Medicine, Cheonan, Republic of Korea; 11 Department of Preventive Medicine, Dankook University College of Medicine, Cheonan, Republic of Korea; 12 Department of Psychology, College of Public Services, Dankook University, Cheonan, Republic of Korea; 13 Environmental Health Center, Dankook University Medical Center, Cheonan, Republic of Korea; 14 Department of Preventive Medicine, College of Medicine, The Catholic University of Korea, Seoul, Republic of Korea; 15 Department of Preventive Medicine and Heavy Metal, Exposure Environmental Health Center, Dong-A University College of Medicine, Busan, Republic of Korea; 16 Department of Pediatrics, Dong-A University College of Medicine, Busan, Republic of Korea; 17 Department of Pediatrics, Inha University School of Medicine, Incheon, Republic of Korea; 18 Environmental Health Center for Allergic Diseases, Inha University Hospital, Incheon, Republic of Korea; 19 The Environmental Health Center, Jeju National University College of Medicine, Jeju, Republic of Korea; 20 Department of Preventive Medicine, Jeju National University College of Medicine, Jeju, Republic of Korea; 21 Department of Microbiology and Immunology, Jeju National University College of Medicine, Jeju, Republic of Korea; 22 Department of Obstetrics and Gynecology, Kangwon National University Hospital, Kangwon National University School of Medicine, Chuncheon, Republic of Korea; 23 Department of Pediatrics, Korea University Anam Hospital, Seoul, Republic of Korea; 24 Environmental Health Center, Korea University Anam Hospital, Seoul, Republic of Korea; 25 Department of Pediatrics, Kosin University Gospel Hospital, Kosin University School of Medicine, Busan, Republic of Korea; 26 Department of Pediatrics, Samsung Medical Center, Sungkyunkwan University School of Medicine, Seoul, Republic of Korea; 27 Environmental Health Center for Atopic Diseases, Samsung Medical Center, Sungkyunkwan University School of Medicine, Seoul, Republic of Korea; 28 Environmental Health Center, Seoul National University College of Medicine, Seoul, Republic of Korea; 29 Institute of Environmental Medicine, Seoul National University Medical Research Center, Seoul, Republic of Korea; 30 Department of Obstetrics and Gynecology, Seoul National University Hospital, Seoul, Republic of Korea; 31 Department of Pediatrics, Seoul National University Hospital, Seoul, Republic of Korea; 32 Department of Occupational and Environmental Medicine, Soonchunhyang University Gumi Hospital, Gumi, Republic of Korea; 33 Environmental Health Center, Soonchunhyang University Gumi Hospital, Gumi, Republic of Korea; 34 Department of Pediatrics, Daegu Catholic University School of Medicine, Daegu, Republic of Korea; 35 Department of Occupational Health, Daegu Catholic University, Kyongsan, Republic of Korea; 36 Taean Environmental Health Center, Taean, Republic of Korea; 37 Department of Preventive Medicine, Dankook University College of Medicine, Cheonan, Republic of Korea; 38 Environmental Health Center, University of Ulsan College of Medicine, Ulsan, Republic of Korea; 39 Department of Occupational and Environmental Medicine, Ulsan University Hospital, University of Ulsan College of Medicine, Ulsan, Republic of Korea

**Keywords:** Computational biology and bioinformatics, Developmental biology, Health care, Risk factors

## Abstract

Exposure to heavy metals such as lead, cadmium, and mercury poses serious health risks to pregnant women because of their high toxicity. In this study, we investigated the associations of heavy metal exposure with birth outcomes of Korean infants. Data of 5,215 women between 2015 and 2019 were analyzed. This study was part of the Korean Children’s Environmental Health (Ko-CHENS) study. Linear regression and logistic regression analyses were used to examine effects of concentrations of lead, cadmium, and mercury on birth weight, small for gestational age, and large for gestational age after adjusting for maternal age groups, parity, infant sex, education, income, smoking, drinking, body mass index, stillbirth, premature birth, diabetes, hypertension, and gestational diabetes. Besides adjusting for these covariates, each metal was mutually adjusted to estimate birth weight and large for gestational age status. Maternal cadmium concentrations during early pregnancy (β =  − 39.96; 95% confidence interval (CI): − 63.76, − 16.17) and late pregnancy (β =  − 37.24; 95% CI − 61.63, − 12.84) were significantly associated with birth weight. Cadmium levels during early pregnancy (adjusted OR = 0.637; 95% CI 0.444, 0.912) were also associated with large for gestational age status. Our findings suggest that prenatal cadmium exposure, even at a low level of exposure, is significantly associated with low birth weight.

## Introduction

Lead, mercury, and cadmium are highly toxic metals associated with extensive environmental contamination and significant health problems. In particular, lead and mercury are highly toxic to fetuses because they can easily cross the blood-placental barrier, while cadmium can only partially cross it^1^. Previous studies have explored effects of lead, cadmium, and mercury on fetal growth outcomes, including small for gestational age status and low birth weight^2–12^.

Heavy metal toxicology can interfere with fetal cell division and differentiation. For example, lead exposure can interfere with calcium deposition in bones during fetal development^13^. Suboptimal fetal growth can result from prenatal cadmium exposure^14^. Methylmercury (MeHg) can adversely affect fetal growth by inhibiting the antioxidant system and increasing free radical production^15^.

A number of epidemiological studies have shown that harmful effects on birth outcomes can significantly impact morbidity and disability in early childhood^16^ and lead to health problems in adulthood, such as respiratory disorders and cardiovascular diseases^16,17^. Low infant birth weight has been associated with several chronic health consequences such as diabetes mellitus, and obesity in adulthood^18^. The aim of this study was to investigate the associations between heavy metal (lead, cadmium, and mercury) exposure during early pregnancy, late pregnancy and at birth with birth outcomes, such as birth weight, small for gestational age and large for gestational age. Although several studies from developed countries have examined lead, cadmium, and mercury exposure in relation to birth outcomes^2,7–9^, our study is significant for having one of the largest samples.

## Methods

### Study population

**Figure 1:**
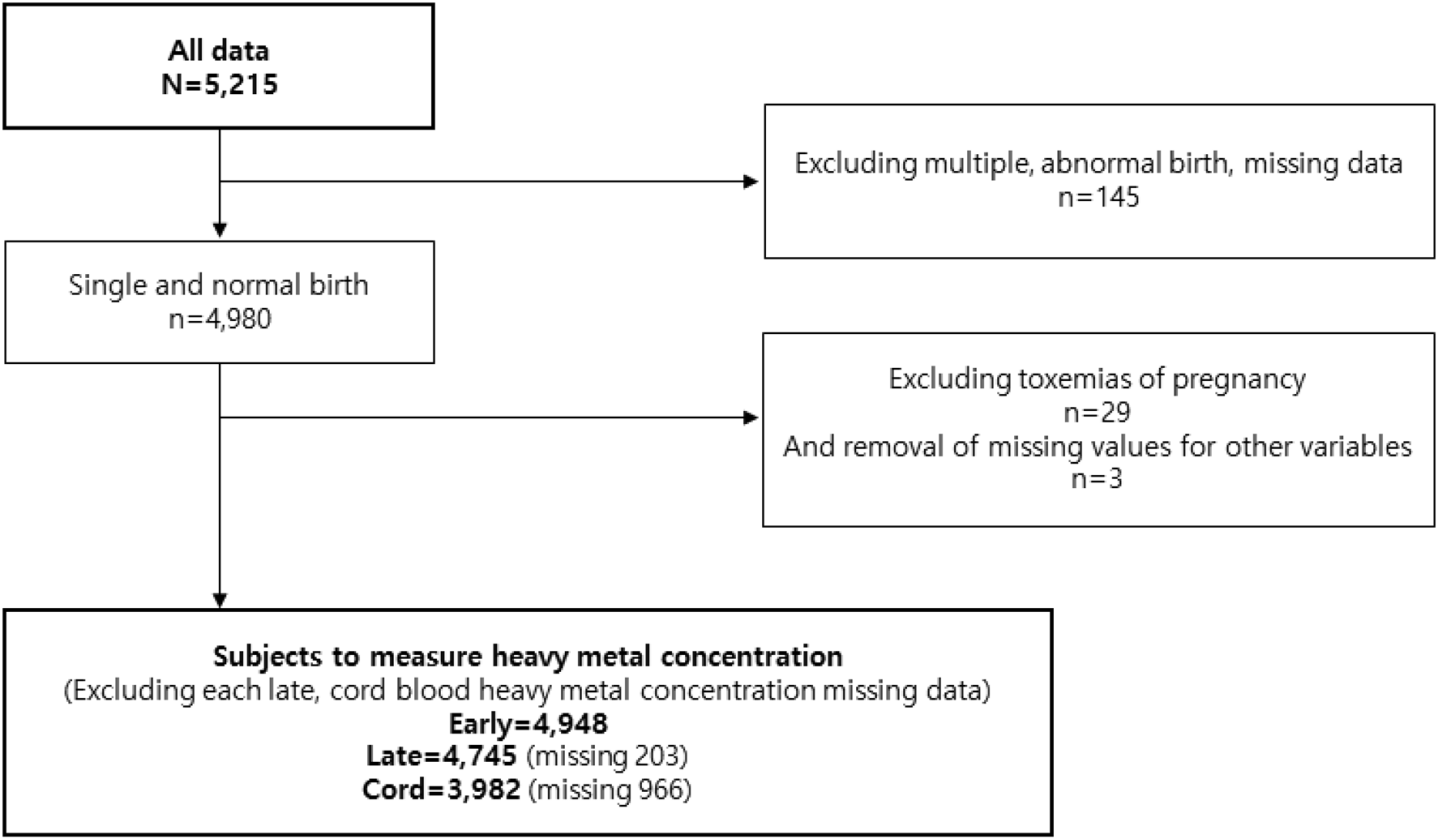
Flowchart showing the selection process of final participants from the Korean Children’s Environmental Health Study (Ko-CHENS) to be included in this study.

This research was a component of the Korean Children’s Environmental Health (Ko-CHENS) Study, which was launched in 2015 with funding from the Ministry of the Environment and the National Institute of Environmental Research to study environmental diseases in children^19^. This study used data collected from a total of 5215 pregnant women from 2015 to 2019. Exclusion criteria for this study were: multiple or abnormal births (n = 145), toxemia of pregnancy (n = 29), and missing covariates (n = 3). Finally, heavy metal concentrations were measured for a total of 4948 women during early pregnancy and 4745 (missing 203) women during late pregnancy. Heavy metal concentrations were also measured for 3982 (missing 966) cord blood samples (Fig. 1). All subjects and/or their legal guardian(s) in this study provided written informed consent. This study was approved by the Institutional Review Board of Kangwon National University Hospital (KNUH-2021-10-003). This study was conformed to the tenets of the Declaration of Helsinki.

### Measurement methods for lead, cadmium, and mercury

Venous blood samples were obtained from participants during early pregnancy (12–20 weeks) and late pregnancy (> 28 weeks) upon outpatient visits. Vacuum blood collection tubes containing sodium ethylenediaminetetraacetic acid were used to collect whole blood samples (Vacutainer®, Beckton & Dickson, Franklin Lakes, NJ, USA). After storing samples in a refrigerator, they were transferred to the laboratory for lead, mercury, and cadmium measurements^20^. Blood metal levels were measured using an Agilent 7900 inductively coupled plasma mass spectrometer (ICP-MS) (Agilent Technologies, Santa Clara, CA, USA). Lead, cadmium, and mercury levels in blood samples were detected at 0.009 μg/dL, 0.05 μg/L, and 0.10 μg/L, respectively. In addition, to handle the limit of detection (LOD) effect we used LOD analysis method, LOD/$\sqrt{2}$, for each batch to improve research measurement accuracy of lead, cadmium and mercury^21^.

### Birth weight, small for gestational age, and large for gestational age determinations

Birth outcomes such as birth weight and perinatal medical information were collected from prenatal care and delivery clinic medical charts. Other health outcomes were measured using questionnaire surveys, medical utilization databases, and health checkup databases. Birth weight was the first weight of the baby. It was taken soon after birth. If an infant’s weight was measured at less than the 10th percentile, it was classified as small for gestational age (SGA). If it was measured greater than the 90th percentile, it was termed large for gestational age (LGA). In this study, a chart suggested by Fenton that was consistent with the World Health Organization growth standard was used^22^.

### Statistical analysis

Multiple linear regression analysis was performed to evaluate the association between prenatal heavy metal exposure and birth weight. Multiple logistic regression analysis was performed to calculate the odds ratio (OR) and 95% confidence interval (CI) for evaluating effects of heavy metal concentrations on SGA and LGA. Statistical model was adjusted for maternal age, parity, infant sex, education, income, smoking, drinking, body mass index (BMI), still birth, premature birth, diabetes, hypertension, and gestational diabetes. The main reason for choosing these variables was their influence on birth outcomes. According to various ethnic studies, maternal smoking correlates with reduced birthweight and low birthweight prevalence among different ethnic groups^23,24^. It has also been shown that maternal age affects birth weight. Low-birth-weight infants are more likely to be born to younger and older mothers^25^. Additionally, infants with low birth weight are also more likely to be preterm births^26^. Besides adjusting for these covariates, a mutually adjusted linear regression model for lead, cadmium, and mercury was used to estimate the association of heavy metal exposure with birth weight. Each metal was also mutually adjusted by a logistic regression model to explore the relationship of heavy metal exposure with LGA. All statistical analyses were performed using SAS 9.4 (SAS Institute Inc., Cary, NC, USA).

## Results

More than 95% of the samples were collected from pregnant women aged 30 years and older (early pregnancy, 96.9%; late pregnancy, 97%; and cord blood, 97.2%). Nearly three-quarters of the women had a normal BMI prior to pregnancy (early and late pregnancy, 73.4%; cord blood, 73.7%). Approximately 76% of the women reported studying in universities. Nearly half of them had a family income of $1500 to $3000. More than 85% of the women had never smoked. Very few reported current alcohol consumption (early pregnancy, 1.7%; late pregnancy and cord blood, 1.8%). It was found that 1.1% of early and late pregnancy groups and 1% of the cord blood group had hypertension and 1.8% of all women had gestational diabetes. Approximately 25% of early and late pregnancy groups and cord blood group reported stillbirth. In birth outcomes, proportions of SGA and LGA were about 12% and 3%, respectively, for all three sample types. The mean birth weight was 3222.2 g (SD = 443.4 g) in the early pregnancy group, 3240.6 g (SD = 405.4 g) in the late pregnancy group, and 3249.9 g (SD = 399.8 g) in the cord blood group (Table 1).

**Table 1:** General characteristics of the study population.

	Early	Late	Cord
	n = 4948	n = 4745	n = 3982
	n (%) or mean ± sd	n (%) or mean ± sd	n (%) or mean ± sd
Maternal age categories			
20–29	154 (3.1)	144 (3.0)	112 (2.8)
30–34	1176 (23.8)	1141 (24.1)	915 (23.0)
34–39	2365 (47.8)	2270 (47.8)	1919 (48.2)
40 +	1253 (25.3)	1190 (25.1)	1036 (26.0)
Parity			
0	3436 (69.4)	3286 (69.3)	2767 (69.5)
1 +	1512 (30.6)	1459 (30.8)	1215 (30.5)
Infant sex			
Male	2533 (51.1)	2432 (51.2)	2028 (50.9)
Female	2415 (48.8)	2313 (48.8)	1954 (49.1)
Education			
Middle & high	600 (12.1)	569 (12.0)	494 (12.4)
University	3745 (75.7)	3591 (75.7)	3018 (75.8)
Graduate school +	603 (12.2)	585 (12.3)	470 (11.8)
Family income (dollars)			
< $1500	326 (6.6)	316 (6.7)	274 (6.9)
$1500 ~ $3000	2464 (49.8)	2358 (49.7)	2026 (50.9)
≥ $3000	2158 (43.6)	2071 (43.7)	1682 (42.2)
Smoking status			
Current	27 (0.6)	26 (0.6)	26 (0.7)
Former	612 (12.4)	585 (12.3)	496 (12.5)
Never	4309 (87.1)	4134 (87.1)	3460 (86.9)
Drinking status			
Current	86 (1.7)	83 (1.8)	72 (1.8)
Former	3734 (75.5)	3584 (75.6)	3027 (76.0)
Never	1128 (22.8)	1078 (22.7)	883 (22.2)
BMI group			
Underweight	532 (10.8)	510 (10.8)	420 (10.6)
Normal	3631 (73.4)	3481 (73.4)	2934 (73.7)
Obese	785 (15.9)	754 (15.9)	628 (15.8)
Stillbirth	1253 (25.3)	1208 (25.5)	995 (25.0)
Hypertension	56 (1.1)	53 (1.1)	39 (1.0)
Diabetes	33 (0.7)	31 (0.7)	24 (0.6)
Gestational diabetes	91 (1.8)	86 (1.8)	71 (1.8)
Preterm birth (< 37 weeks)	262 (5.3)	199 (4.2)	148 (3.7)
Birth outcomes			
Birth weight (grams)	3222.2 ± 443.4	3240.6 ± 405.4	3249.9 ± 399.8
SGA	628 (12.7)	605 (12.8)	504 (12.7)
LGA	145 (2.9)	139 (2.9)	119 (3.0)

Data are presented as numbers (%) or mean ± standard deviation.

*SGA* small for gestational age, *LGA* large for gestational age, *BMI* body mass index.

Maternal blood lead concentration was 0.74 ± 0.42 µg/dL in the early pregnancy group, 0.70 ± 0.58 µg/dL in the late pregnancy group, and 0.55 ± 0.33 µg/dL in the cord blood group. Cadmium levels were 0.62 ± 0.31 µg/L and 0.70 ± 0.32 µg/L in early and late pregnancy groups, respectively, and 0.24 ± 0.12 µg/L in the cord blood group. Mercury levels were 2.37 ± 1.26 µg/L and 1.95 ± 1.03 µg/L in early and late pregnancy groups, respectively, and 3.62 ± 1.99 µg/L in the cord blood group (Table 2).

**Table 2:** Heavy metal concentrations (lead, cadmium, mercury).

	Mean ± SD	n of < LOD	Min	5%	10%	25%	50%	75%	90%	95%	Max
Lead (μg/dL)											
Early	0.74 ± 0.42	606	0.25	0.25	0.25	0.48	0.67	0.92	1.21	1.42	6.11
Late	0.70 ± 0.58	736	0.25	0.25	0.25	0.44	0.62	0.84	1.12	1.35	24.78
Cord	0.55 ± 0.33	0	0.13	0.13	0.20	0.33	0.49	0.70	0.93	1.11	4.16
Cadmium (μg/L)											
Early	0.62 ± 0.31	335	0.23	0.23	0.23	0.42	0.57	0.76	1.00	1.19	3.53
Late	0.70 ± 0.32	755	0.23	0.23	0.37	0.49	0.66	0.86	1.07	1.26	3.71
Cord	0.24 ± 0.12	0	0.13	0.13	0.13	0.13	0.23	0.29	0.36	0.40	1.95
Mercury (μg/L)											
Early	2.37 ± 1.26	1463	0.14	1.01	1.19	1.55	2.07	2.84	3.87	4.72	12.02
Late	1.95 ± 1.03	374	0.06	0.88	1.02	1.31	1.72	2.31	3.09	3.72	19.81
Cord	3.62 ± 1.99	0	0.43	1.53	1.81	2.37	3.16	4.30	5.77	7.24	23.51

*LOD* limit of detection.

In the multiple linear regression model, after adjusting for variables in multivariate regression, birth weight showed significant negative associations with cadmium level in early pregnancy (adjusted OR = -39.96 (95% CI − 63.76, − 16.17; P = 0.0010), cadmium level in late pregnancy (adjusted OR = − 37.24 (95% CI − 61.63, − 12.84; P = 0.0028), and lead level in late pregnancy (adjusted OR = − 23.80 (95% CI − 44.50, − 3.10; P = 0.0243). However, cord blood lead level (adjusted OR = 30.02 (95% CI 10.38, 49.65; P = 0.0027) showed a significant positive correlation with birth weight (Table 3).

**Table 3:** Multiple regression of log-transformed lead, cadmium, and mercury levels.

	Birth weight	
	Beta (95% CI)	*p*-value
Early		
Lead (μg/dL)	− 19.89 (− 41.13, 1.34)	0.0664
Cadmium (μg/L)	− 39.96 (− 63.76, − 16.17)	0.0010*
Mercury (μg/L)	− 0.28 (− 23.81, 23.24)	0.9810
Late		
Lead (μg/dL)	− 23.80 (− 44.50, − 3.10)	0.0243*
Cadmium (μg/L)	− 37.24 (− 61.63, − 12.84)	0.0028*
Mercury (μg/L)	− 11.54 (− 35.38, 12.30)	0.3428
Cord		
Lead (μg/dL)	30.02 (10.38, 49.65)	0.0027*
Cadmium (μg/L)	− 14.03 (− 42.40, 14.33)	0.3322
Mercury (μg/L)	− 11.06 (− 35.94, 13.80)	0.3830

Adjusted for maternal age group, parity, infant sex, education, income, smoking, drinking, BMI group, stillbirth, premature birth, diabetes, hypertension, and gestational diabetes.

*p-value < 0.05.

After controlling for potential confounding factors, Fenton LGA status showed significant positive associations with early pregnancy cadmium level (adjusted OR = 0.637 (95% CI 0.444, 0.912; P = 0.0139) and mercury level (adjusted OR = 1.439 (95% CI 1.010, 2.051; P = 0.0441) as well as cord blood lead level (adjusted OR = 1.443 (95% CI 1.035, 2.012; P = 0.0305). However, lead, cadmium, and mercury levels in early pregnancy, late pregnancy, and cord blood samples were not significantly associated with Fenton SGA status (Table 4).

**Table 4:** Multiple logistic regression of log-transformed lead, cadmium, and mercury levels.

	SGA		LGA	
	Odds ratio (95% CI)	*p*-value	Odds ratio (95% CI)	*p*-value
Early				
Lead (μg/dL)	1.059 (0.899, 1.246)	0.4937	0.871 (0.626, 1.212)	0.4134
Cadmium (μg/L)	1.066 (0.887, 1.282)	0.4956	0.637 (0.444, 0.912)	0.0139*
Mercury (μg/L)	0.909 (0.758, 1.090)	0.3030	1.439 (1.010, 2.051)	0.0441*
Late				
Lead (μg/dL)	1.151 (0.975, 1.359)	0.0958	0.956 (0.687, 1.330)	0.7889
Cadmium (μg/L)	1.044 (0.858, 1.271)	0.6681	0.790 (0.535, 1.166)	0.2349
Mercury (μg/L)	1.052 (0.868, 1.276)	0.6047	1.381 (0.952, 2.004)	0.0893
Cord				
Lead (μg/dL)	0.942 (0.804, 1.105)	0.4646	1.443 (1.035, 2.012)	0.0305*
Cadmium (μg/L)	1.244 (0.991, 1.562)	0.0595	0.795 (0.502, 1.258)	0.3270
Mercury (μg/L)	1.048 (0.856, 1.284)	0.6473	1.202 (0.813, 1.778)	0.3567

Adjusted for maternal age group, parity, infant sex, education, income, smoking, drinking, BMI group, stillbirth, premature birth, diabetes, hypertension, and gestational diabetes.

*SGA* small for gestational age, *LGA* large for gestational age.

*p-value < 0.05.

In the mutually adjusted linear regression model, when lead and other confounding factors were adjusted, a significant negative association between maternal blood cadmium level with birth weight was seen in early pregnancy (beta = − 36.62, 95% CI − 61.20, − 12.05; P = 0.0035) and late pregnancy (beta = − 32.65, 95% CI − 57.65, − 7.65; P = 0.0105). After adjusting for cadmium level along with other confounding factors, birth weight showed a statistically significant positive association with cord blood lead level (beta = 31.26, 95% CI 11.53, 50.98; P = 0.0019). Also, after adjusting for mercury and other confounding factors, cadmium levels in early (beta = − 40.71, 95% CI − 64.74, − 16.68; P = 0.0009) and late (beta = − 36.28, 95% CI − 61.01, − 11.55; P = 0.0040) pregnancy showed significant negative associations with birth weight. However, birth weight showed a significant positive association with lead level in cord blood (beta = 32.52, 95% CI 12.58, 52.46; P = 0.0014) (Table 5).

**Table 5:** Mutually adjusted linear regression model of log-transformed lead, cadmium, and mercury levels.

	Birth weight (grams)			
	Beta (95% CI)	*p*-value	Beta (95% CI)	*p*-value
Early				
Cadmium (μg/L)	− 36.62 (− 61.20, − 12.05 )^1^	0.0035	− 40.71 (− 64.74, − 16.68)^2^	0.0009
Late				
Cadmium (μg/L)	− 32.65 (− 57.65, − 7.65)^1^	0.0105	− 36.28 (− 61.01, − 11.55)^2^	0.0040
Cord				
Lead (μg/dL)	31.26 (11.53, 50.98)^3^	0.0019	32.52 (12.58, 52.46)^2^	0.0014

^1^Adjusted for lead, maternal age group, parity, infant sex, education, income, smoking, drinking, BMI group, stillbirth, premature birth, diabetes, hypertension, and gestational diabetes.

^2^Adjusted for mercury, maternal age group, parity, infant sex, education, income, smoking, drinking, BMI group, stillbirth, premature birth, diabetes, hypertension, and gestational diabetes.

^3^Adjusted for cadmium, maternal age group, parity, infant sex, education, income, smoking, drinking, BMI group, stillbirth, premature birth, diabetes, hypertension, and gestational diabetes.

In mutually adjusted logistic regression, after adjusting for lead along with other confounding factors, LGA showed significant positive associations with early pregnancy cadmium level (beta = 0.643, 95% CI 0.444, 0.932; P = 0.0198) and early pregnancy mercury level (beta = 1.502, 95% CI 1.047, 2.154; P = 0.0271). When cadmium level was adjusted along with other confounding factors, early pregnancy mercury level (beta = 1.542, 95% CI 1.078, 2.205; P = 0.0177) and cord blood lead level (beta = 1.471, 95% CI 1.053, 2.056; P = 0.0236) showed significant positive associations with LGA. After adjusting for mercury and other confounding factors, LGA was associated with maternal blood early pregnancy cadmium level (beta = 0.601, 95% CI = 0.419, 0.863; P = 0.0058) and cord blood lead level (beta = 1.421, 95% CI 1.014, 1.989; P = 0.0410) (Table 6).

**Table 6:** Mutually adjusted logistic regression model of log-transformed lead, cadmium, and mercury levels.

	LGA			
	Beta (95% CI)	*p*-value	Beta (95% CI)	*p*-value
Early				
Cadmium (μg/L)	0.643 (0.444, 0.932)^1^	0.0198	0.601 (0.419, 0.863)^2^	0.0058
Mercury (μg/L)	1.502 (1.047, 2.154)^1^	0.0271	1.542 (1.078, 2.205)^3^	0.0177
Cord				
Lead (μg/dL)	1.471 (1.053, 2.056)^3^	0.0236	1.421 (1.014, 1.989)^2^	0.0410

*LGA* large for gestational age.

^1^Adjusted for lead, maternal age group, parity, infant sex, education, income, smoking, drinking, BMI group, stillbirth, premature birth, diabetes, hypertension, and gestational diabetes.

^2^Adjusted for mercury, maternal age group, parity, infant sex, education, income, smoking, drinking, BMI group, stillbirth, premature birth, diabetes, hypertension, and gestational diabetes.

^3^Adjusted for cadmium, maternal age group, parity, infant sex, education, income, smoking, drinking, BMI group, stillbirth, premature birth, diabetes, hypertension, and gestational diabetes.

## Discussion

This study examined adverse effects of prenatal exposure to heavy metals on birth outcomes in a Korean population. We investigated heavy metal concentrations in early and late pregnancy and in cord blood. After controlling for confounders, maternal cadmium concentrations in early and late pregnancy were significantly associated with low birth weights of infants.

The mean maternal blood cadmium concentration was 0.62 μg/L in early pregnancy, 0.70 μg/L in late pregnancy, and 0.24 μg/L in cord blood. The mean maternal and cord blood cadmium concentrations in this study were higher than those reported in the United Kingdom (mean 0.56 μg/L in early pregnancy) in 2016^3^, in Australia (mean 0.54 μg/L in late pregnancy) in 2013^27^, in an eastern China study conducted in 209 pregnant women in late pregnancy (mean 0.48 μg/L) and cord blood (mean 0.09 μg/L)^10^ in 2014, in North Carolina, USA (mean 0.46 mg/L in late pregnancy) in 2014^5^, in South Africa (mean 0.25 μg/L in late pregnancy and 0.27 μg/L in cord blood) in 2015^6^; and in Norway in second-trimester smokers (Geometric mean, GM = 0.26 μg/L) and non-smokers (GM = 0.15 μg/L) in 2011^28^. However, our study found lower cadmium levels in maternal blood in late pregnancy (mean 0.98 μg/L) and cord blood (mean 0.78 μg/L) than those in a Saudi Arabian study in 2014^7^. A number of factors, such as the number of subjects and the type of covariates used, might have contributed to such differences in cadmium levels in above-mentioned results from developed and developing countries.

The present study also showed lower cadmium concentrations than those in the Korean multi-center prospective birth cohort MOCEH study. However, our study found trends similar to those in the MOCEH study (late pregnancy maternal blood: 1.51 μg/L vs. early pregnancy maternal blood: 1.41 μg/L vs. cord blood 0.67 μg/L)^29^. Blood cadmium concentrations during late pregnancy were higher than those during early pregnancy. They were the lowest in cord blood (late pregnancy maternal blood: 0.70 μg/L vs. early pregnancy maternal blood: 0.62 μg/L vs. cord blood 0.24 μg/L). These observations were consistent with another study (late pregnancy maternal blood: 0.98 μg/L vs. cord blood: 0.78 μg/L)^7^. Although cadmium levels in our study were lower than those in the above studies, cadmium is harmful even at low concentrations^30^. Moreover, we found that maternal and cord blood cadmium levels, even when they were low, were more strongly associated with an increased risk of low birth weight and LGA status than lead and mercury levels.

This study found that blood lead concentrations were particularly low (0.74 μg/dL in early pregnancy, 0.70 μg/dL in late pregnancy, and 0.55 μg/dL in cord blood) compared to those in a study in China of 209 pregnant women (third-trimester maternal blood lead GM = 3.95 μg/dL and cord blood GM = 3.16 μg/dL) in 2014^10^, another study in China of 252 mother-infant pairs (maternal blood lead GM = 3.53 μg/dL and cord blood lead level GM = 2.92 μg/dL)^4^, and a study in Saudi Arabia (late pregnancy maternal blood lead concentration mean = 2.89 μg/dL and cord blood mean = 2.55 μg/dL) in 2014^7^. However, some studies have found similar levels, including a study in Norway (second-trimester maternal blood lead GM = 0.75 μg/dL) in 2011^28^ and a study in the United States (midterm pregnancy blood lead GM = 0.7 μg/dL) in 2018^31^. Some studies showed lower concentrations than ours, including an Australian study (late pregnancy maternal blood mean = 0.5 μg/L) in 2013^27^ and a Puerto Rican study (maternal blood GM = 0.33 μg/dL)^9^.

Maternal blood mercury concentrations in our study (early pregnancy maternal blood 2.37 μg/L and late pregnancy maternal blood 1.95 μg/L) were greater than those in studies conducted in Norway (mean = 1.2 μg/L)^28^, China (GM = 0.84 μg/L)^32^, Australia (mean = 0.83 μg/L)^27^, and the United States (GM = 0.6 μg/L)^31^, but lower than in those in studies conducted in Greenland (mean = 16.8 μg/L)^33^ and Saudi Arabia (mean = 3.00 μg/dL)^7^. Mercury concentrations in our study were also lower than those in a Korean study (early pregnancy maternal blood GM = 3.67 μg/L, late pregnancy maternal blood GM = 3.30 μg/L and cord blood GM = 5.53 μg/L) that was a part of the Mothers and Children’s Environmental Health Study performed between 2006 and 2008 on 417 Korean women and newborns^34^. Despite having lower mercury levels than the above Korean study, our study had a large sample size and showed a significant association between mercury and LGA status. We also accounted for lead and cadmium concentrations, whereas the above Korean study only focused on mercury concentrations.

Lead concentrations (early pregnancy maternal blood 0.74 vs. late pregnancy maternal blood 0.70 vs. cord blood 0.55 μg/dL) and mercury concentrations (early pregnancy maternal blood 2.37 vs. late pregnancy maternal blood 1.95 vs. cord blood 3.63 μg/L) in this study were consistent with those of the MOCEH study (lead: early pregnancy maternal blood GM = 1.30 vs. late pregnancy maternal blood GM = 1.20 vs. cord blood 0.92 μg/dL; mercury: early pregnancy maternal blood GM = 3.29 vs. late pregnancy maternal blood GM = 3.05 vs. cord blood GM = 5.10 μg/L)^29^. In our study, blood lead concentrations during late pregnancy were lower than those in early pregnancy. As lead can move from blood into bones during pregnancy, physiological factors such as increases in plasma estrogen concentrations might have contributed to this decrease^35,36^. Total blood mercury levels (GM) in late pregnancy were lower than those in early pregnancy. Previous studies have shown the same trend^37,38^. This decrease of blood mercury level during late pregnancy is due to the diluting effect of increased plasma volume^39^.

Several studies have examined effects of heavy metal exposure on newborn anthropometrics^7,10,11^. Among these, cadmium was found to have the most profound impact on several birth outcomes, although birth outcomes showed no correlation with lead or mercury concentrations in the same population^7^. Since heavy metals cause physiological immaturity during pregnancy and early life, they can pose a serious threat to fetal and infant health^40^. The effects of cadmium on apoptosis, oxidative stress, reactive oxygen species, and deoxyribonucleic acid (DNA) repair can be attributed to its toxicity^40^. Cadmium may also affect growth in fetuses by affecting 11beta-hydroxysteroid dehydrogenase type 2 activity^41^. During pregnancy, exposure to cadmium has been linked to decreased birth weights and premature births, and elevated levels of placental cadmium resulting from maternal exposure to industrial waste or tobacco smoke have been associated with decreased progesterone biosynthesis by the placental trophoblast^42^. Also, A number of potential mechanisms can contribute to cadmium-induced fetal growth restriction (FGR), including hypoxia in the fetus, disturbed fetoplacental zinc homeostasis, and reduced blood flow to the uterus and placenta^42,43^. Previous studies have shown a link between prenatal cadmium exposure and low birth weight^5,10^. A total of 408 mother-infant pairs in Hubei Province, China provided evidence of a positive association between maternal cadmium exposure and the risk of infant preterm low birth weight (PLBW)^44^. An earlier cross-sectional study of 209 pregnant women in Eastern China observed that maternal blood cadmium levels were inversely related to birth weight (r = − 0.22; P = 0.03)^10^. Another study of 1027 pregnant women in the United States reported that high maternal blood cadmium levels (≥ 0.50 μg/L) were negatively associated with birth weight percentile for gestational age and positively associated with SGA (OR = 1.71; 95% CI 1.10, 2.64)^5^. Consistent with these results, our findings also indicated that high maternal blood cadmium levels (early pregnancy 0.62 μg/L and late pregnancy 0.70 μg/L) were inversely associated with birth weight, whereas they showed no association with SGA.

Our analysis demonstrated that cord blood lead level was positively associated with birth weight. Although we observed a statistically significant positive association, it could be due to the impact of other factors, such as maternal nutrition that may influence birth weight. As such, nutritional intake was not considered in this study, although it could have an impact on low birth weight^45^. Therefore, we could not completely exclude the influence of dietary intake on metal exposure measurements^46^. One previous study has also detected a positive association between birth weight and nickel^9^. Likewise, another study reported a non-significant positive association between cadmium level and birth weight (maternal blood, β = 87.0, 95% CI − 63.1–237.0; cord blood, β = 55.0, 95% CI − 108.2–218.3)^47^. However, previous studies showed a significant inverse association between lead and birth weight^4,48^ or no association^11,12^.

This study had several strengths. First, this study had a prospective Korean birth cohort design with extensive information on potential confounders. The main strength of this study was its large sample size. Moreover, heavy metals were estimated at two time points, early pregnancy and late pregnancy, to provide accurate associations between heavy metal levels and outcomes. However, this study also had some limitations. First, this study only focused on three major heavy metals. The presence of other toxic heavy metals might have affected the main result. For example, one study showed that selenium level was associated with newborn birth weight and that increased selenium intake might decrease cord blood cadmium concentrations^10^. However, the majority of previous studies, including ours, demonstrated that cadmium was linked to lower birth weight^2,7^. Second, genetic information that could be correlated with birth outcomes was not included in the present study^49^.

## Conclusion

Our results suggest that low levels of prenatal exposure to cadmium, lead, and mercury might affect birth outcomes. This study provides further support for the need to reduce cadmium exposure among pregnant women as much as possible. Although the effect of heavy metal exposure on birth outcomes might be small, their consequences might not be negligible. Further studies on effects of prenatal exposure to a variety of metals present in the environment on birth outcomes are needed.

## Data Availability

Data were deposited in the National Institute of Environment Research. For access, please contact the NIER and corresponding author.

## Abbreviations

SGA: Small for gestational age
LGA: Large for gestational age
BMI: Body mass index
OR: Odds ratio
SD: Standard deviation
GM: Geometric mean
CI: Confidence interval
MOCEH: Mothers and Children’s Environmental Health Study
Ko-CHENS: Korean Children’s Environmental Health Study
